# Bio‐Inspired Multi‐Mode Pain‐Perceptual System (MMPPS) with Noxious Stimuli Warning, Damage Localization, and Enhanced Damage Protection

**DOI:** 10.1002/advs.202004208

**Published:** 2021-03-08

**Authors:** Fali Li, Shuang Gao, Ying Lu, Waqas Asghar, Jinwei Cao, Chao Hu, Huali Yang, Yuanzhao Wu, Shengbin Li, Jie Shang, Meiyong Liao, Yiwei Liu, Run‐Wei Li

**Affiliations:** ^1^ CAS Key Laboratory of Magnetic Materials and Devices Ningbo Institute of Materials Technology and Engineering Chinese Academy of Sciences Ningbo 315201 P. R. China; ^2^ Zhejiang Province Key Laboratory of Magnetic Materials and Application Technology Ningbo Institute of Materials Technology and Engineering Chinese Academy of Sciences Ningbo 315201 P. R. China; ^3^ College of Materials Science and Opto‐Electronic Technology University of Chinese Academy of Sciences Beijing 100049 P. R. China; ^4^ Department of Mechanical Materials and Manufacturing Engineering The University of Nottingham Ningbo China Ningbo 315100 P. R. China; ^5^ School of Future Technology University of Chinese Academy of Sciences Beijing 100049 P. R. China; ^6^ National Institute for Materials Science 1‐1 Namiki Tsukuba Ibaraki 305‐0044 Japan

**Keywords:** bio‐inspired, damage protection, e‐skin, noxious stimuli, pain‐perceptual

## Abstract

The multi‐mode pain‐perceptual system (MMPPS) is essential for the human body to perceive noxious stimuli in all circumstances and make an appropriate reaction. Based on the central sensitization mechanism, the MMPPS can switch between different working modes and thus offers a smarter protection mechanism to human body. Accordingly, before injury MMPPS can offer warning of excessive pressure with normal pressure threshold. After injury, extra care on the periphery of damage will be activated by decreasing the pressure threshold. Furthermore, the MMPPS will gradually recover back to a normal state as damage heals. Although current devices can realize basic functions like damage localization and nociceptor signal imitating, the development of a human‐like MMPPS is still a great challenge. Here, a bio‐inspired MMPPS is developed for prosthetics protection, in which all working modes is realized and controlled by mimicking the central sensitization mechanism. Accordingly, the system warns one of a potential injury, identifies the damaged area, and subsequently offers extra care. The proposed system can open new avenues for designing next‐generation prosthetics, especially make other smart sensing systems operate under complete protection against injuries.

## Graphical abstract

Inspired by the comprehensive protection mechanism of humans, a multi‐mode pain‐perceptual system is developed for prosthetic protection. Before an injury, the system can provide early warning. After the injury, damaged area is located and extra protection is realized, during which minor pressure can induce pain. Furthermore, the pressure threshold gradually restores to normal as the damaged area heals.

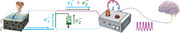

## 1 Introduction

The somatosensory system decodes various types of stimuli and endows the human body with a remarkable ability of object recognition, texture discrimination, sensory‐motor feedback, and social exchange.^[^ ^1^ ^]^ Furthermore, the human body has developed a multi‐mode pain‐perceptual system (MMPPS) for discriminating noxious stimuli in all circumstances (before injury, on injury, and after injury) and making appropriate reactions.^[^ ^2^ ^]^ Multifunctional electronic skins (e‐skins) have been developed by learning the structure and working mechanism of the somatosensory system.^[^ ^3^, ^4^, ^5^, ^6^, ^7^, ^8^ ^]^ However, the e‐skin cannot distinguish noxious stimuli and work safely without the MMPPS. Therefore, it is necessary to study the working mechanism of MMPPS for designing noxious stimuli‐resistant e‐skin. In human skin, sensors related to innocuous stimuli are distributed in the dermal layer, while nociceptors are distributed in the epidermal layer. Before injury, pressure sensors (such as Pacinian corpuscles) can warn one of excessive pressure based on normal pressure threshold. In the case of an injury, the nociceptors respond to damage and the body reacts evasively. After facing an injury, the central sensitization mechanism is activated, which decreases the pressure threshold of the periphery of damage.^[^ ^2^, ^22^ ^]^ This mechanism will offer extra care for tiny pressure, which can induce pain. Furthermore, the MMPPS will gradually restore to normal state as the damage heals. In short, the coordination of multiple sensors and the nervous system is the basis for the MMPPS and this can significantly help design e‐skins.^[^ ^1^, ^2^ ^]^

Recently, two strategies were proposed for designing the pain‐perceptual system (PPS) for e‐skin.^[^ ^9^, ^10^, ^11^, ^12^ ^]^ The first strategy includes a pressure sensor that distinguishes noxious stimuli based on a specific threshold. For example, Osborn et al. designed a pressure ‐sensor‐based nociceptor, that activated when the pressure reaches 150 kPa and guides the prosthetics to perform invasive action.^[^ ^11^ ^]^ This strategy is effective only before experiencing a mechanical injury. The second strategy is based on the change in resistance of the nanomaterial after experiencing mechanical damage. For example, the resistance of the mixture of liquid metal (LM) and Polydimethylsiloxane (PDMS) decreases dramatically in the event of an injury.^[^ ^14^ ^]^ Based on this factor, Markvicka et al. designed a damage‐detection sensor and realized the locating of damage. These two strategies effectively offer dangerous warnings (in advance of injury) and locate the damage (site of injury).^[^ ^12^ ^]^ However, unlike PPMS, current bio‐inspired pain‐perceptual devices cannot offer e‐skin protection after injury; the e‐skin is much more fragile after injury. In addition, researchers have recently proposed the use of memristors or transistors to imitate the neural characteristics of artificial synapses.^[^ ^15^, ^16^, ^17^, ^18^, ^19^ ^]^ By combining such artificial synaptic devices, we can find a way to integrate damage‐detection sensors with pressure sensors for reproducing MMPPS on the e‐skin.

Herein, the MMPPS is designed for the e‐skin based on the coordination between the pressure sensors and electronic damage‐detection sensors (e‐nociceptors), and the working mode of the MMPPS is controlled by an artificial synapse. The pressure sensor distinguishes the noxious stimuli based on different pressure thresholds in different working modes. The damage‐detection sensor detects the mechanical damage based on the decrease in resistance due to the rupturing of the liquid metal particles (LMP). Furthermore, different working modes of MMPPS are realized by the switching of the resistance of the artificial synapse (Pt/Nb: SrTiO_3_ heterojunction memristor).^[^ ^20^ ^]^ Before an injury, the artificial synapse is in the high resistance state, which helps the system operate in the normal state, and only a high pressure (≥150 kPa^[^ ^11^ ^]^) can induce pain. After an injury, the damage‐detection sensor locates the damaged area and guides prosthetics to take evasive actions. At the same time, the artificial synapse is switched to the low resistance state, which creates a central sensitization effect. After an injury, the pressure threshold is decreased, and even a minor pressure (1.5 kPa, minutes after the damage) acting on the damaged area is sufficient to trigger pain. During the healing process, the central sensitization mechanism gradually decays as the resistance of the artificial synapse increases. To make the smart prosthetics compatible with the human neural system, the Izhikevich neural framework model was applied, which converts the obtained resistance signal into a neuromorphic signal.^[^ ^11^, ^21^ ^]^ Finally, we demonstrated the performance of MMPPS on a robotic hand, which verifies that the MMPPS can offer constant (before, during and after an injury) protection to the e‐skin.

## 2 Results and Discussion

### 2.1 Working Mechanism of Human MMPPS

**Figure 1:**
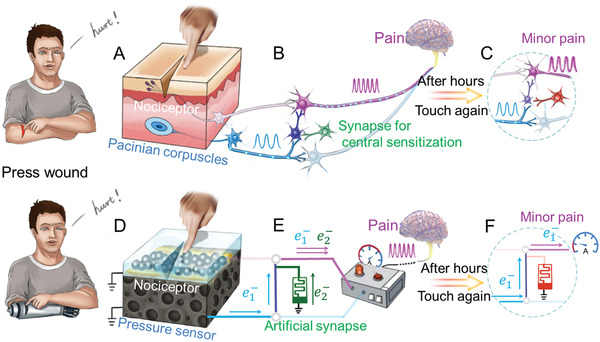
MMPPS of the human body and smart prosthetics. In the human body, touching the damage slightly can trigger pain. A) Human skin consists of nociceptors and pressure sensors (Pacinian corpuscles), which connects to the brain separately. B) After injury, the central sensitization mechanism is activated by the neuron (green color refers to activation) between two neural pathways. Pacinian corpuscle's signal transfers into the pathway for nociceptor and trigger pain in brain. C) As the damage heals, the central sensitization mechanism decays gradually, and the pressure withstanding ability of damage recovers simultaneously. D) Bio‐inspired e‐skin integrated with LM‐based damage‐detection sensor (LM nerve endings and inter‐deposited LM‐based e‐cells) and pressure sensor (porous PDMS coated with graphene). E) The combined output of the damage‐detection sensor and pressure sensor is transferred to the signal processing unit, which can convert the electrical signal to neuromorphic signals. After injury, the artificial synapse attains a low resistance state (green), which activates the central sensitization mechanism. Consequently, the signal from the pressure sensor will influence the combined output, and finally, pain is triggered even after a slight touch. F) After several hours, the artificial synapse gradually shifts to a high‐resistance state (red), which decays the central sensitization mechanism, and the pressure capability of the damage recovers gradually.

The human skin consists of nociceptors and pressure sensors, which are distributed in the epidermal and dermal layers, respectively (**Figure** **1**A).^[^ ^1^, ^2^, ^22^ ^]^ The pressure sensors (e.g., Pacinian corpuscles) sense the pressure on the skin, while nociceptors (consisting of free nerve endings) are responsible for damage detection. Nociceptors and pressure sensors are connected to the brain through separate neural pathways (Figure 1B). An additional neuron exists between these pathways that controls the central sensitization mechanism and decides the working modes of the MMPPS.^[^ ^2^ ^]^

Before injury, pressure sensor distinguishes noxious stimuli and transmits them to the human brain via neural pathway. High pressure can influence the neural signal, and brain will control the body to make evasive action. At this time, the nociceptor remains silent and will not interfere with the working of the pressure sensor^[^ ^2^ ^]^ (i.e., no information transfer from neural pathway of pain to pressure).

In advance of an injury, the pressure sensor perceives noxious stimuli and transmits it to the brain through a neural pathway. A high pressure can influence the neural signal, and the body acts evasively based on this signal. During this time, the nociceptor is inactive and does not interfere with the pressure sensor^[^ ^2^ ^]^ (i.e., no information transfer from the neural pathway of pain to pressure).

At the time of the injury, the ruptured skin cells release various inflammatory signals (such as histamine, glutamate, ATP).^[^ ^2^ ^]^ The pain that is felt after the injury is due to the impact of these inflammatory signals on surrounding nociceptors. At this moment, the nociceptors transmit high‐frequency signals (representing pain) to the brain, which influences the body to take evasive actions.

After the injury, the damaged area needs several days to heal. During this time, the area surrounding the damaged part is very fragile, and even a minor pressure may cause another injury. However, due to the central sensitization mechanism, neural signals are transferred from the pathway of the pain to that of the pressure, through the inter‐located neuron ^[^ ^2^ ^]^ (present between two pathways in Figure 1B). Consequently, even a slight pressure on the damaged area generates pain, which improves the protection on the healing area.

The central sensitization mechanism decays gradually with the healing of the damaged area, and this is mainly due to the decay of signal that is transferred between the two neural pathways. Therefore, the pressure withstanding capability of the damaged area gradually increases. The capability of the damaged area to withstand normal pressure is restored once it completely heals (Figure 1C).

The cooperative interaction between various sensors and the neural system of the human body contributes to the efficient functioning of MMPPS. This system operates before, during, and after an injury, thus enabling the human body to deal with the changing environments.

### 2.2 Working Mechanism of Bio‐inspired MMPPS

By imitating the structure of human skin, the damage‐detection sensor and pressure sensor were fabricated in the dermal and epidermal layers, respectively (Figure 1D). A film of LMP is used in the e‐cell due to which the damage sensor shows resistance when a particular area is damaged. The pressure sensor was fabricated by coating the porous PDMS with graphene. These sensors are connected to the testing unit through separate circuits. In all working modes, a resistance lower than the threshold resistance (*R*_th_) is chosen as the signal for generating pain. The artificial synapse (Pt/Nb: SrTiO_3_ heterojunction memristor) is responsible for activating the central sensitization mechanism and controlling the working mode of this system. In addition, to make the MMPPS compatible with the human body, the Izhikevich neural framework model is used, which translates the resistive signal of sensors into neuromorphic signals (Figure 1E).

Before an injury, the pressure sensor monitors the pressure applied on e‐skin, and its resistance changes with pressure. When the pressure is higher than 150 kPa, the testing unit (Figure 1E) detects a resistance lower than *R*_th_ and send pain signals to the brain. Evasive actions are taken by prosthetics to avoid danger.

**Figure 2:**
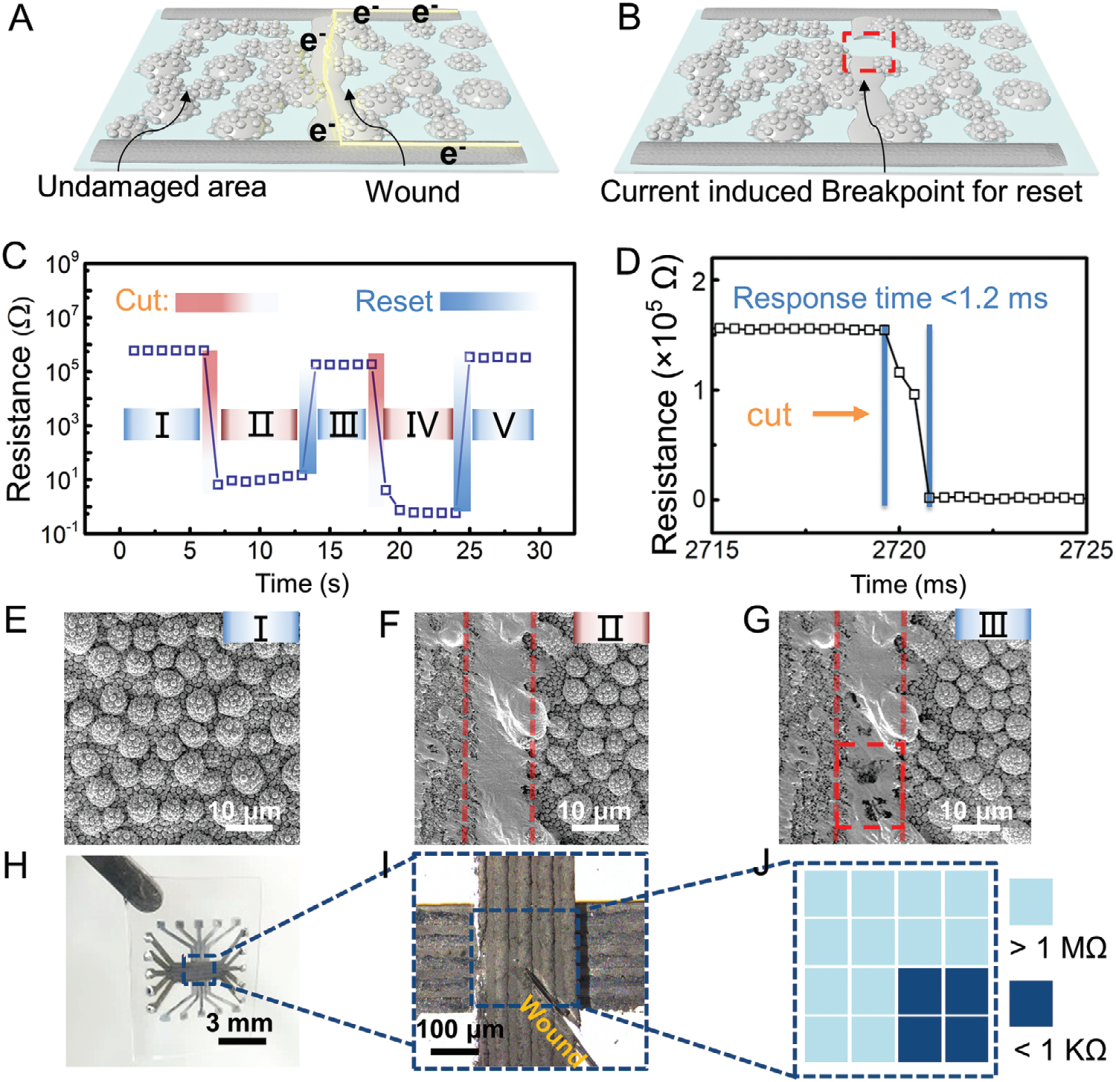
Performance of bio‐inspired damage sensor. A) Schematic of the structure of damage‐detection sensor, wherein the LM‐based e‐cells are deposited between e‐nerve endings. And injuring the damage‐detection sensor results in the formation of LM conductive path. B) LM conductive path breaks when applying reset current. C) The change in resistance of damage‐detection sensor during the repeated cutting and resetting. D) Response time of the damage‐detection sensor evaluated by cutting the sensor at very high speed (about 5 m s^−1^). SEM images of the damage sensor: E) before injury, F) after injury, and G) after reset. Formation and breakage of the LM conductive path are highlighted in (F) and (G), respectively. H) Optical image of damage‐detection sensor array with crossbar structure. I) Microscope image of damage‐detection sensor showing an enlarged view of its damage, and the width of each cell is 50 µm. J) The resistances of every sensor in this array are shown with different colors to locate the damage.

In the event of an injury, the ruptured e‐cells (electronic cells) on the e‐skin release the LM that they contain, which dramatically decreases the resistance of damage‐detection sensors. After being detected by the testing unit, it sends a high‐frequency (conveying pain) neural signal to the brain.^[^ ^21^ ^]^ At the same time, the damage‐detection sensor is reset by large current for monitoring subsequent dangerous stimuli. (Details are shown in **Figure** **2**).

**Figure 3:**
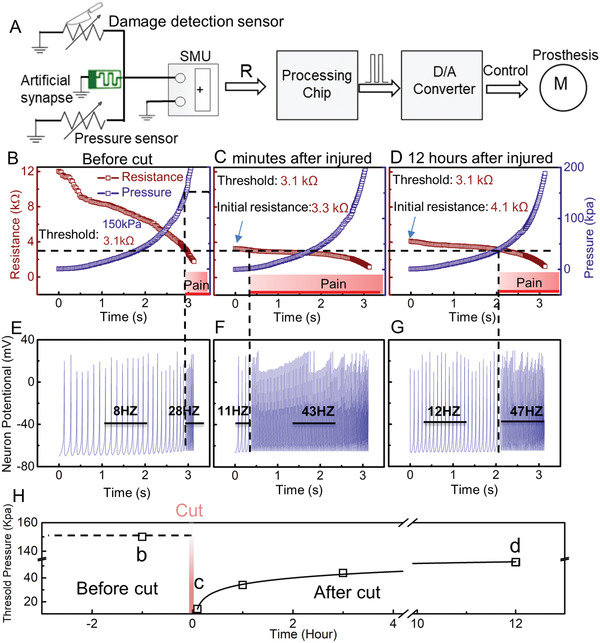
Performance of bio‐inspired MMPPS. A) Block diagram of bio‐inspired MMPPS, wherein the damage‐detection and pressure sensor are placed in a parallel combination to the artificial synapse. The output of source measurement units (SMU) enters a signal processing chip integrated with the Izhikevich neural framework model and LabVIEW program. The LabVIEW program evaluates the resistance signal and controls prosthetics, while the Izhikevich neural framework model converts the resistance signal to neuromorphic signal. Variation in resistance signal with respect to pressure acting on the damage. B) Before the injury, 150 kPa is considered as the threshold for healthy skin to produce pain.^[^ ^11^ ^]^ Under this pressure, *R*_SMU_ is 2.9 kΩ, which is further used as a threshold for pain generation in (C) and (D). C) After injured for several minutes, with central sensitization activated, the *R*_SMU_ reaches 2.9 kΩ only at 1.5 kPa and cause pain. D) After injured for 12 h, owing to the decay of central sensitization, *R*_SMU_ is higher than 2.9 kΩ until 40 kPa. E–G) Based on the Izhikevich neuron framework, the data in (B)–(D) is transferred to the neuromorphic signal that can be recognized by human nerve. Before the pain is generated, the neuromorphic signal following the mode of regular spiking (low frequency), and after that, it shifts to fast spiking (high frequency). H) The *P*_th_ is different under different working modes (before and after injured), Points b, c, d in the curve are correspond to (B), (C), and (D).

After the injury, the central sensitization mechanism is activated by switching the resistance of the artificial synapse to a low level (slightly higher than *R*_th_). As the artificial synapse and sensors are connected in parallel, the results (without pressure) from the testing unit are slightly higher than *R*_th_ according to the parallel combination rule. When a slight pressure is applied on the pressure sensor, it sends a high‐frequency neural signal to the brain, and the results from the testing unit are lower than *R*_th_. (Details are shown in **Figure** **3**).

During the healing process of e‐skin, the resistance of the artificial synapse gradually increases.^[^ ^20^, ^23^ ^]^ According to the parallel combination rule, the results (without pressure) from the testing unit increase based on the resistance recovery of the artificial synapse. Therefore, the pressure withstanding capability of the damage gradually increases. The damaged area restores its capability of withstanding normal pressure when it heals completely (Figure 1F). (Details are presented in Figure 3).

### 2.3 Preparation and Performance of Bio‐inspired Damage Sensor

Gallium‐based liquid metals are widely used owing to their unique combination of non‐toxicity, fluidity, and metallic properties.^[^ ^24^ ^]^ When the LM is exposed to air, a thin oxide skin forms on its surface spontaneously, which enables the LM to maintain various structures.^[^ ^25^, ^26^ ^]^ This oxide layer prevents the flow of metal until the externally applied pressure is higher than the surface yield stress of the oxide skin.^[^ ^24^ ^]^ Moreover, the LM can be easily shaped into LMPs with variable sizes.^[^ ^14^, ^27^ ^]^ Therefore, LMP (e‐cells) is used to imitate the function of cells that activate the damage‐detection sensor when broken. Accordingly, the structure is similar to that of cell fluids, which can activate nerve endings during injury.

The bio‐inspired damage‐detection sensor is composed of e‐nerve endings (electronic nerve endings) and inter‐deposited LM e‐cells, as shown in Figure 2A. The LM e‐nerve endings were prepared by printing LM paste on a PDMS substrate through stencil printing. The LM paste was prepared by doping LM with Cu particles, which also increased the viscosity of the LM. LM e‐cells are densely deposited between the LM wires via physical vapor deposition. As the LM does not wet the substrate PDMS, e‐cells can be easily fabricated during physical vapor deposition. By controlling the deposition time, the diameter of the e‐cells was set as 10 µm (at the same level as the skin cell). As there is no oxide layer in a vacuum, the connected e‐cells merge into larger e‐cells during the deposition. Consequently, we can obtain a film whose e‐cells are separated from one another (Figure S2A–C, Supporting Information). In the next step, this film is exposed to air for oxide layer formation. This oxide layer can stabilize the first layer of e‐cells, and the latter serves as a substrate for the second layer of e‐cells. By controlling the deposition time when fabricating the second layer of e‐cells, we can obtain a film of e‐cells one another (Figure S2, Supporting Information).

Before injury, e‐cells are electrically separated owing to the insulating e‐cell membrane (gallium oxide layer). When an injury occurs, the membrane ruptures and causes the outflow of e‐cell fluid, which eventually leads to the formation of an LM conductive path between the nerve endings (Figure 2A). Furthermore, as the e‐skin may work in a complex environment, an injured e‐skin may experience another injury. To ensure that the e‐skin can detect multiple injuries, we reset the damage‐detection sensor by breaking its conductive path that is formed during the previous injury. Researchers have reported that an LM thin film breaks when a relatively high current is applied. This process is called the electromigration induced break‐up (EMIB).^[^ ^28^, ^29^ ^]^ Based on this phenomenon, the reset current is applied to break the LM conductive path in the damaged area and restore the original resistance (Figure 2B and Figure S4, Supporting Information).

Figure 2C shows the change in resistance of the damage‐detection sensor (*R*_d_) during the repetitive cutting and resetting process. Initially (normal condition), *R*_d_ is at the MΩ level (zone I), which drops below 100 Ω (zone II) due to injury. Following this, *R*_d_ jumps back to the MΩ level immediately after the reset step (zone III), followed by another drop (zone IV) that results from the second injury. Zone V illustrates another recovery of resistance to the MΩ level through the reset step. Figure 2D shows the response time of the damage‐detection sensor, which is evaluated by cutting the damage‐detection sensor with a scalpel at high speed (≈5 m s^−1^). Our sensor showed an ultrafast response time (<1.2 ms). Figure 2E–G shows the corresponding SEM images of the damage‐detection sensor: before injury (zone I), after cutting (zone II), and after resetting (zone III). Accordingly, the damage sensor exhibited high resistance because the e‐cells are intact (Figure 2E). *R*_d_ drops rapidly due to the formation of the LM conductive path during injury (Figure 2F). The conductive path breaks when the reset current is applied, and the resistance recovers thereafter (Figure 2G).

To locate the damage, an array of damage‐detection sensor was fabricated with a crossbar structure, and its optical images are shown in Figure 2H. In Figure 2I, the enlarged view of the injured sensor array is clearly presented with a damage at its bottom right. The resistance of each unit in the damage sensor array is tested and shown in Figure 2J, wherein the dark blue color represents low resistance, and the light blue color represents high resistance. As the sneak‐path problem exists during the readout of the resistance of the sensor array. This structure is not precise enough and can only get an approximate position of the damaged area. By integrating switch devices in future work, one can overcome the sneak‐path problem and locate the damaged area precisely. In short, the damaged area can be located by analyzing the distribution of resistance.

### 2.4 Preparation and Performance of Bio‐inspired MMPPS

The bio‐inspired MMPPS is realized by the cooperation of the damage‐detection sensor and the pressure sensor, and this is coordinated by an artificial synapse. Figure 3A shows the block diagram of the bio‐inspired MMPPS, wherein the source measurement unit (SMU) is placed parallel to the damage‐detection sensor (*R*_d_), pressure sensor (*R*_p_), and artificial synapse (*R*_s_). The resistance measured by the SMU changes according to Equation (1). The output of the SMU is sent to a signal processing unit, which, in this case, is a computer integrated with LabVIEW program and Izhikevich neural framework model. The LabVIEW program guides prosthetics about taking evasive actions. Furthermore, the Izhikevich neural framework can convert the resistance signal into a neuromorphic signal.

(1) $$\frac{1}{R_{\mathrm{SMU}}}=\frac{1}{R_{d}}\;+\frac{1}{R_{s}}+\;\frac{1}{R_{p}}$$

Figure 3B–D shows the change in *R*_SMU_ with respect to the applied pressure on e‐skin. When the resistance is smaller than the threshold (*R*_th_, 2.9 kΩ in this system), the resistance is recognized as a signal of pain by the processing unit. In addition, the resistance signal is converted into neuromorphic signals by the Izhikevich neural framework model (Figure 3E–G). The specific working modes of the bio‐inspired MMPPS are shown in the following paragraphs.

**Before injury**, pain is generated when excessive pressure is applied onto the skin. The pressure threshold (*P*_th_) is defined as the critical pressure at which the human body feels pain. According to previous studies, 150 kPa was chosen as the *P*_th_.^[^ ^11^ ^]^ As there was no damage to the skin, both the damage‐detection sensor and artificial synapse exhibited high resistance (MΩ level), and *R*_p_ decreased from 12 to 2 kΩ as the pressure increased from 0 to 200 kPa. In this state, *R*_p_ is in the order of kΩ, while *R*_s_ and *R*_d_ remain at the MΩ level. According to the law of parallel summation, *R*_SMU_ is affected mainly by the pressure sensor, and other devices can be neglected (Equation (2)). Figure 3B shows the variation of *R*_SMU_ with respect to the pressure acting on the uninjured e‐skin. When the pressure equals *P*_th_, *R*_SMU_ exhibits a corresponding resistance of 2.9 kΩ (equal to *R*_th_). Figure 3E shows the corresponding neuromorphic signal, which is calculated from the resistance signal using the Izhikevich neural framework model. When the *R*_SMU_ is higher than *R*_th_, the frequency of the neuromorphic signal is 8 Hz (regular spiking), and it becomes 28 Hz when the *R*_SMU_ is below *R*_th_ (fast spiking).

(2) $$\frac{1}{R_{\mathrm{SMU}}}\approx \frac{1}{R_{p}}$$

**When an injury occurs**, *R*_d_ decreases instantly from MΩ to a value lower than 1 kΩ (due to the formation of the LM conductive path), which then causes a sudden drop in *R*_SMU_ and triggers a signal of pain. Figure S5 (Supporting Information) shows the variation of *R*_SMU_ when cutting the e‐skin. When cut by a scalpel, the *R*_SMU_ decreases dramatically from 12 to 0.1 kΩ (Equation (3)). The frequency of the corresponding neuromorphic signal increases from 6 to 70 Hz. After the injury, the following steps are taken to activate the central sensitization. First, the reset current is applied to the damage‐detection sensor to break the injury‐induced LM conductive path. This reset current shifts the *R*_d_ back to high resistance state (MΩ) and allows the damage‐detection sensor to monitor subsequent injuries (if any). Second, a voltage for activating the central sensitization mechanism is applied to the artificial synapse, and its resistance is switched from MΩ to kΩ (details shown in Figure S6C, Supporting Informaiton). According to the parallel combination law, as *R*_d_ is at the MΩ level, *R*_s_ and *R*_p_ remain at the kΩ level, and the *R*_SMU_ is influenced mainly by the artificial synapse and pressure sensor (Equation (4)). In the subsequent working modes, the artificial synapse plays the role of a synapse in the human body. It makes the e‐skin more sensitive to pressure and converts the signal of even a slight touch into pain.

(3) $$\frac{1}{R_{\mathrm{SMU}}}\approx \frac{1}{R_{d}}+\;\frac{1}{R_{p}}$$

(4) $$\frac{1}{R_{\mathrm{SMU}}}\approx \frac{1}{R_{s}}+\;\frac{1}{R_{p}}$$

**After injury for several minutes**, the human skin becomes much more fragile as compared to the normal state.^[^ ^2^ ^]^ Therefore, pain signals should be triggered at pressures below the normal value. The human body activates the central sensitization mechanism to ensure that the injured skin is protected. We have developed this central sensitization mechanism in the bio‐inspired MMPPS by tuning the *R*_s_ values. By decreasing *R*_s_ (from 2.5 MΩ to 4.1 kΩ)_,_ the zero‐pressure resistance of *R*_SMU_ is close (slightly higher) to *R*_th_. Figure 3C shows a variation of *R*_SMU_ with respect to the pressure on the damaged area several minutes after the injury (Equation (4)). A slight touch on the damaged area (1.5 kPa) ensures that the *R*_SMU_ is lower than *R*_th_. Figure 3F shows the corresponding neuromorphic signal calculated from the resistance signal in Figure 3C using the Izhikevich model. The frequency of the neuromorphic signal remains at 11 Hz below the pressure of 1.5 kPa, and it becomes 43 Hz when the external pressure exceeds this minor pressure. This imitated the central sensitization mechanism and offered extra care to the injured e‐skin. During the subsequent healing process, the *R*_s_ gradually shifted back to the high‐resistance state (Figure S6D, Supporting Information). Accordingly, the central sensitization mechanism decays gradually,^[^ ^2^ ^]^ and this can control the level of extra protection to injured e‐skin with different healing times.

**After injury for 12 h**, the damaged area healed to a certain extent, and its pressure tolerance also recovered by a certain extent. By applying a self‐healing polymer, researchers have realized the self‐healing ability of the e‐skin.^[^ ^13^, ^30^, ^31^ ^]^ Furthermore, we imitated the recovery of pressure capability by maximizing the resistance decay characteristics of the artificial synapse (Figure S5D, Supporting Information). Figure 3D shows the variation of *R*_SMU_ with respect to the pressure applied to the damaged area. Applying a medium pressure (40 kPa) to the damaged area can cause the *R*_SMU_ to be lower than *R*_th,_ and thus induce pain. Compared to the value observed after a few minutes after the injury, the *P*_th_ increases significantly in this case. This occurs because the SMU is testing the parallel combination of the artificial synapse and the pressure sensor (Equation (4)). Due to the gradual increase in *R*_s_, the zero‐pressure resistance of *R*_SMU_ increased from 3.3 to 4.1 kΩ. Therefore, a higher pressure (compared to the one that was applied minutes after the injury) is needed to ensure that the *R*_SMU_ is lower than *R*_th_. Figure 3G shows the corresponding neuromorphic signal, which is calculated from the resistance signal presented in Figure 3D using the Izhikevich model. The frequency of the neuromorphic signal remains 12 Hz below 40 kPa (new pressure threshold), and it reaches 47 Hz when the external pressure exceeds the medium level.

Figure 3H summarizes the mechanism of the MMPPS under various working modes. Before the injury, the *P*_th_ required to trigger pain was 150 kPa, and the corresponding *R*_th_ of this system was 2.9 kΩ. After the injury, *P*_th_ rapidly drops to 1.5 kPa due to the decrease in *R*_s_. As *R*_s_ increases with time, the threshold also recovers to 40 kPa after 12 h after the injury. Thus, the MMPPS is effectively reproduced on the e‐skin.

### 2.5 Demonstration of Bio‐inspired MMPPS

To clearly demonstrate the operation of the MMPPS, the system was connected to a robotic hand. We fixed the e‐skin (with the damage‐detection sensor and pressure sensor) on the index finger of the robotic hand. The SMU (Keithley 237) reads the resistance and transfer it to the LabVIEW program for further analysis. The LabVIEW program served as the brain throughout this process. It first analyzes the signal and then instructs the motor of the robotic hand to perform the corresponding evasive action. A pressure is applied to the robotic finger with the rubber ring, and the length of the rubber ring indicates the magnitude of the pressure. We selected 12 kΩ as the *R*_th_ of the system (based on the mechanical capability of the robotic hand).

**Figure 4:**
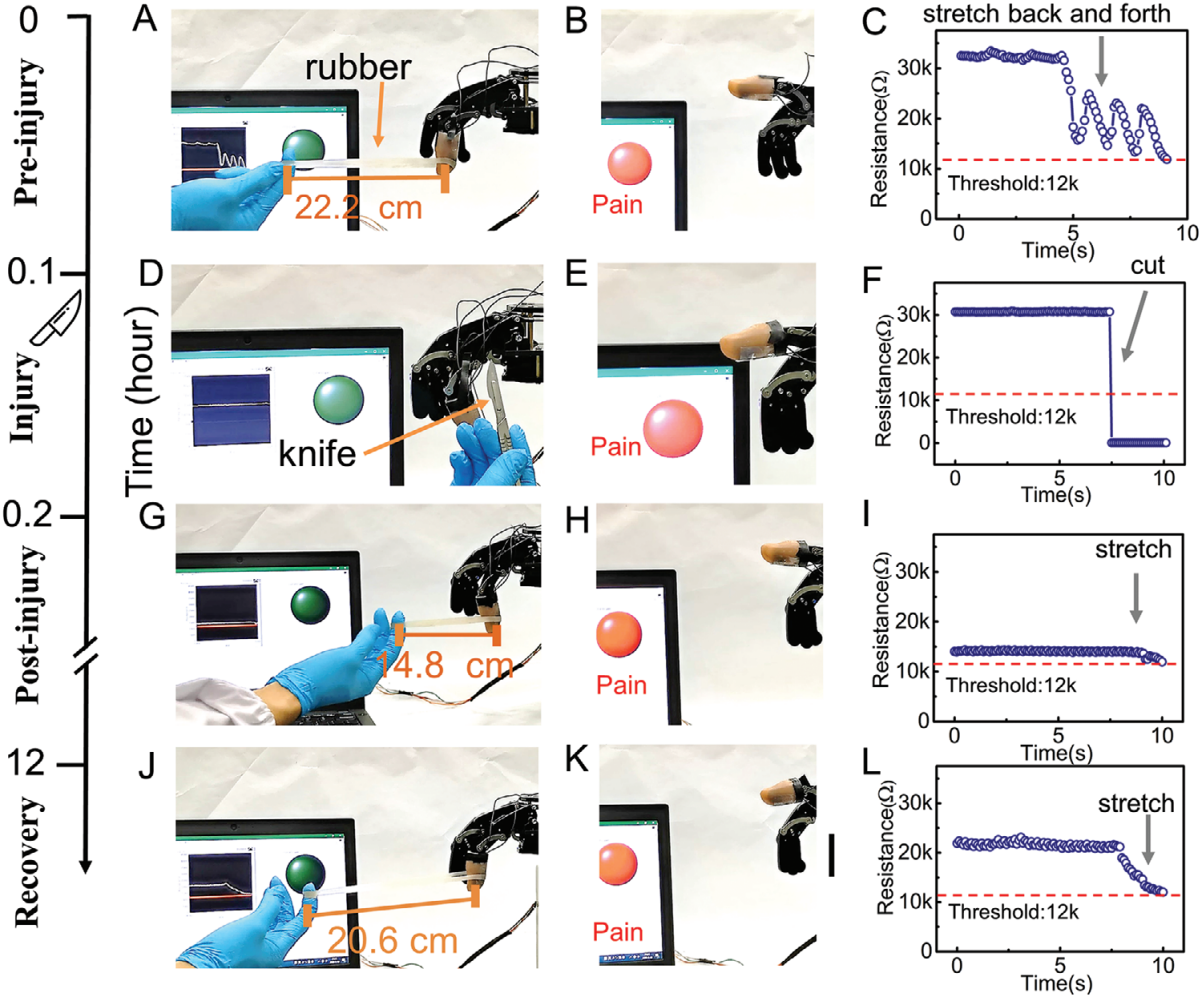
Demonstration of bio‐inspired MMPPS. Sensors are integrated on the robot finger and connected to the artificial synapse. Controlling by a LabVIEW program, 12 kΩ is selected as the threshold resistance for triggering pain (depends on the strength of the robot hand). Working process before the injury. A) Apply pressure on the e‐skin by stretching the O‐shaped rubber between the human finger and the robot finger. Pain triggering when stretching length of rubber ring reaches 22.2 cm, B) Straightening of the finger (evasive action) due to triggered pain, C) Corresponding change in resistance of this system. Working process when being injured. D) Cutting e‐skin with scalpel activates damage‐detection sensor, E) Straightening of the finger due to triggered pain. F) Corresponding resistance drop due to cutting. Working process after injured for minutes. G) Pain triggering at slighter pressure (small stretch length 14.8 cm) due to activated Central sensitization, H) Straightening of the finger due to triggered pain. I) Corresponding change in resistance of the system. Working process after injured for 12 h. J) Pain triggering at medium pressure (medium stretch length 20.6 cm) due to decayed Central sensitization, indicating the restored pressure withstanding capability of damage, K) Straightening of the finger due to triggered pain, L) Corresponding change in resistance of the system.

**Figure** **4**A–C shows the working mode of the bio‐inspired MMPPS. Before the injury, the pressure on the robot fingers is increased by stretching the rubber ring (Figure 4A), and the corresponding changes in *R*_SMU_ are shown in Figure 4C (Equation (2)). When the rubber is stretched to 22.2 cm (original length 12 cm), the *R*_SMU_ is the same as *R*_th_ (12 kΩ). At this time, the LabVIEW program determines that the applied pressure will cause pain, and it instructs the motor to straighten the bent finger (details show in Video S1, Supporting Information).

At the time of the injury, a scalpel is used to cut the e‐skin, and the corresponding resistance change is shown in Figure 4F (Equation (3)). The *R*_SMU_ was high before the injury, but it dropped quickly after the injury. When the LabVIEW program determines that the resistance is lower than the threshold (12 kΩ), it instructs the motor to straighten the bent finger to move the finger away from scalpel (Figure 4D,E). (Details are shown in Video S2, Supporting Information).

Figure 4G–I shows the working mode minutes after the injury. In Figure 4G, the stretched rubber ring is used to apply pressure to the injured e‐skin. Since the central sensitization mechanism is already activated, the corresponding resistance of *R*_SMU_, shown in Figure 4I, is lower than 12 kΩ on the slighter pressure (the rubber ring is stretched to 14.8 cm). When a low *R*_SMU_ is detected by the LabVIEW program, it immediately instructs the motor to straighten the bent fingers of the robotic hand to get away from the rubber ring.

Figure 4J–L shows the working mode 12 h after the injury. After a long healing time, the *R*_s_ decays back to the higher level, which improves the initial resistance of the system. The comparison between Figure 4I,L confirms the improvement in the initial resistance of the system (from 15 to 23 kΩ). Owing to this improvement, the damaged area can restore its capability of withstanding large pressure due to which the system triggers the protective action (Figure 4K) at a large stretching length of 20.6 cm.

## 3 Conclusion

In summary, the human MMPPS is designed from two perspectives: structure imitation of the skin nociceptor and the imitation of the central sensitization mechanism. The damage‐detection sensor is constructed by introducing “liquid metal cells” and “liquid metal nerve endings” to locate mechanical damage. Furthermore, an artificial synapse is introduced to integrate the damage sensor and pressure sensor and control the working mode of the MMPPS. To the best of our knowledge, this is the first report that imitates all working modes of the PPS. Accordingly, (1) before the injury, a danger warning is realized by a pressure sensor. (2) When injured, the damage location is realized by the damage sensor. Subsequently, the central sensitization mechanism is activated by the low resistance state of the artificial synapse. (3) After the injury, a sensitized pressure sensor can offer extra protection to the damage. (4) During healing (if using self‐healing materials), the pressure withstanding capability is restored as central sensitization decays gradually. The demonstration on the robotic hand shows that the MMPPS can deal with the need for noxious stimuli in all situations, especially the enhanced protection of the damaged area. Furthermore, to make the intelligent prosthetics compatible with the human body, the Izhikevich model is implemented, which converts the resistance signal of the sensor into a neuromorphic signal that is compatible with the nervous system. Consequently, the designed MMPPS may open new avenues for the advancement of next‐generation smart sensing systems for protected operations in e‐skins and smart prosthetics.

## 4 Experimental Section

Gallium, indium, and tin (99.99%; Beijing Founde Star Sci. & Technol. Co., Ltd) were mixed in a ratio of 67.5:21.5:10 by mass. Subsequently, the mixture was heated and stirred for 30 min, protected by nitrogen gas at 60 °C to obtain liquid metal Galinstan (Ga68.2, In21.8, Sn10).

Galinstan and copper microparticles (3–5 µm) (ZhongNuo Advanced Material) in a ratio of 17:3 (w/w) were used to increase viscosity.

##### Preparation of Damage Sensor

Substrate preparation: PDMS prepolymer (Sylgard 184, Dow Corning) and curing agent were mixed uniformly at 10:1 (w/w). The uncured PDMS was spin‐coated on the silicon wafer at a speed of 200 rpm and cured at 60 °C for 2 h.

Preparation of liquid metal nerve endings: A mechanical mask (Shenzhen Rigorous Technology Co. Ltd) was used to print copper‐doped Galinstan on a PDMS substrate, which formed a liquid metal electrode array. The width of the hollow part of the mask was 200 microns, and the interval between the hollows was 100 µm. First, the mask was attached to the PDMS substrate, and then a scraper blade was used to spread the copper‐doped Galinstan on the top of the mask. Second, tweezers were used to carefully remove the mask, and thus obtained a liquid metal electrode array with the size same as that of the hollow part of the mask.

LMP film deposition: The electrode array prepared above into the physical vapor deposition equipment (Beijing Techno ZH‐300) was put, and 0.3 mL Galinstan was used as the evaporation material. The evaporation voltage was 2.0 V; the current was 175 A, and the evaporation stopped after 5 min. After the sample was removed from the vacuum chamber, an oxide film was quickly formed on the surface of the LMPs. This was the first layer of “liquid metal cells.” The above sample was placed into the high‐vacuum resistance evaporation equipment again, and the vapor‐deposit for 2 min under the same parameters formed a continuous layer of “liquid metal cells.” Subsequently, the PDMS was used for encapsulation to form a damage‐detection sensor that can sense damage in a single direction (the direction corresponding to the array electrode).

Preparation of the crossbar structure: Two pieces of the above‐mentioned samples on the PDMS were taken, placed them at a 90‐degree cross, and used the PDMS for overall packaging.

##### Preparation of Pressure Sensor

The sugar cubes (taikoo) in a beaker containing uncured PDMS were submerged and then placed the beaker in a vacuum chamber for 1 h. The PDMS‐filled sugar cube was removed from the vacuum chamber and heated at 60 °C for 3 h. The sugar cubes were placed in water and stirred for 1 h to obtain porous PDMS. A 5 mL of alcohol (1% by weight) with carbon powder (ZhongNuo Advanced Material) was dispersed on the porous PDMS and heated at 60 °C for 1 h.

##### Preparation of Artificial Synapse

The fabrication of the artificial synapse was started with a commercially available Nb:SrTiO_3_ (100) single‐crystalline substrate (HEFEI KEJING MATERIALS TECHNOLOGY CO., LTD.), which was 5 × 5 × 0.5 mm^3^ in size with one side polished and had a Nb doping content of 0.7 wt%. Pt electrodes with a thickness of ≈30 nm and a diameter of 0.1–1 mm were deposited on the polished side of the substrate by the electron beam evaporation (ULVAC, MUE‐ECO) with the help of a metal shadow mask.

##### Electrical and Mechanical Characterization

The repeatability of the damage sensor was tested by a Keithley 237 SMU, where the two ends of the damage sensor were electrically connected through copper electrodes. For monitoring the noxious signal, the applied voltage was 0.1 V. While resetting the sensor, the applied current gradually increased from 0 to 100 mA until a sudden increase in resistance. The response time of the damage sensor was tested using the SMU to apply current (1 mA) and the oscilloscope (YOKOGAWA DLM2024) to record the voltage. The resistance properties of the artificial synapse were tested using a semiconductor device parameter analyzer (Agilent B1500A). In the compression and stretching test (Instron 5943), the resistance of the damage sensor was tested by the SMU (Keithley 237) at a speed of 1 mm min^−1^.

##### Demonstration Experiment on the Machine Hand

The damage sensor and pressure sensor were attached to the index finger of the robotic hand (ZL‐robot). Copper wires were used to connect these two sensors with an artificial synaptic device, and SMU (Keithley 237) was used to test the resistance of the system. Both the SMU (Keithley 237) and machine hand were controlled by a program written in a software (NI LabVIEW).

## Conflict of Interest

The authors declare no conflict of interest.

## Supporting information

Supporting Information

Supporting Video 1

Supporting Video 2

Supporting Video 3

Supporting Video 4

## Data Availability

The data that support the findings of this study are available from the corresponding author upon reasonable request.
