# Supplementary material for: Bio‐Inspired Multi‐Mode Pain‐Perceptual System (MMPPS) with Noxious Stimuli Warning, Damage Localization, and Enhanced Damage Protection
Source: Adv Sci (Weinh). 2021 Mar 8;8(10):2004208. doi: 10.1002/advs.202004208 (PMC8132158; doi:10.1002/advs.202004208)
Supplement: Supplementary file 1 — Supporting Information [file ADVS-8-2004208-s003.pdf]

WILEY-VCH

© 2021 Wiley-VCH GmbH

ADVANCED SCIENCE
Open Access

Supporting Information

for *Adv. Sci.,* DOI: 10.1002/advs.202004208

Bio-inspired Multi-mode Pain-perceptual System (MMPPS) with Noxious Stimuli Warning, Damage Localization and Enhanced Damage Protection

Fali Li, Shuang Gao, Ying Lu, Waqas Asghar, Jinwei Cao, Chao Hu, Huali Yang, Yuanzhao Wu, Shengbin Li, Jie Shang, Meiyong Liao, Yiwei Liu, and Run-Wei Li**

Supporting Information

Bio-inspired multi-mode pain-perceptual system (MMPPS) with noxious stimuli warning, damage localization and enhanced damage protection

Fali Li, Shuang Gao, Ying Lu, Waqas Asghar, Jinwei Cao, Chao Hu, Huali Yang, Yuanzhao Wu, Shengbin Li, Jie Shang, Meiyong Liao, Yiwei Liu, and Run-Wei Li**

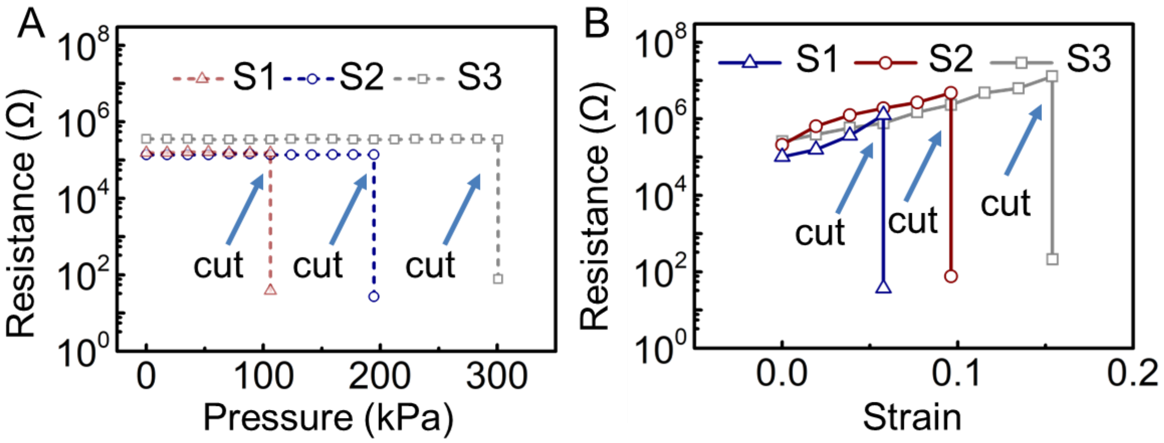

Figure S1. Silent properties of damage sensor. (A) Apply pressure gradually to three samples respectively. The resistance shows no change before injury. (B) Gradually apply strain to three nociceptors and nociceptor's resistance shows no decrease during the deformation process. Only when the sensor is injured will the resistance decrease.

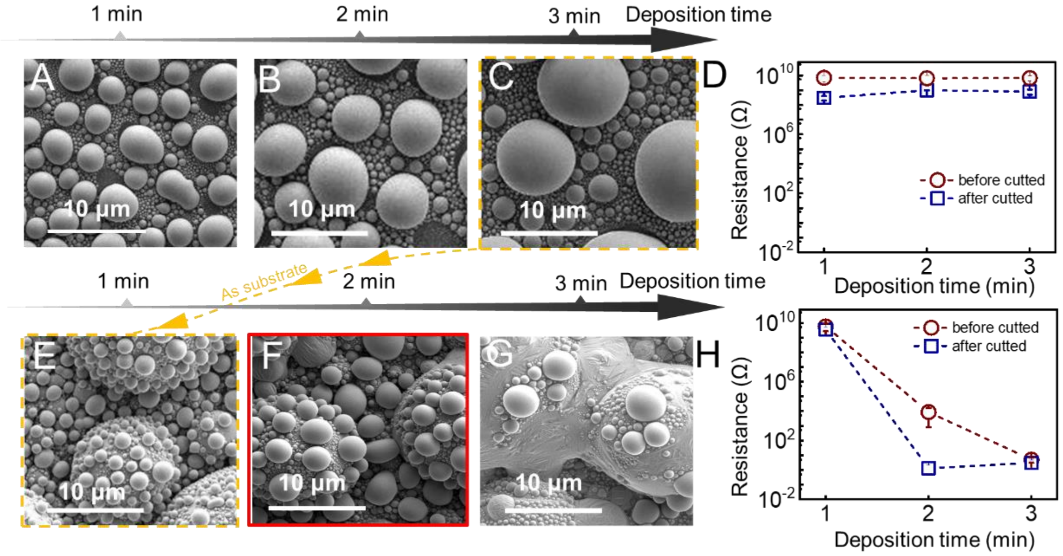

Figure S2. The fabrication and properties of LM e-cell. (A-C) LM deposition on PDMS. Due to the high surface tension, the LM does not wet the substrate. The LM always keeps the shape of the ball and the diameter increases with deposition time. (D) The resistance of the LM film is measured before and after injury which is always in a high level as all LM particles are separated from each other. (E-G) The sample in

D is exposed to air to form an oxide layer (Ga_2O_3) and is deposited with LM again. There is the second layer of LM e-cells formed on the top of the oxide layer. (H) The resistance of the LM film is measured before and after injury. The sample with the first layer of LM which is deposited for 3 minutes and the second layer which is deposited for 2 min shows the best on the on/off ratio.

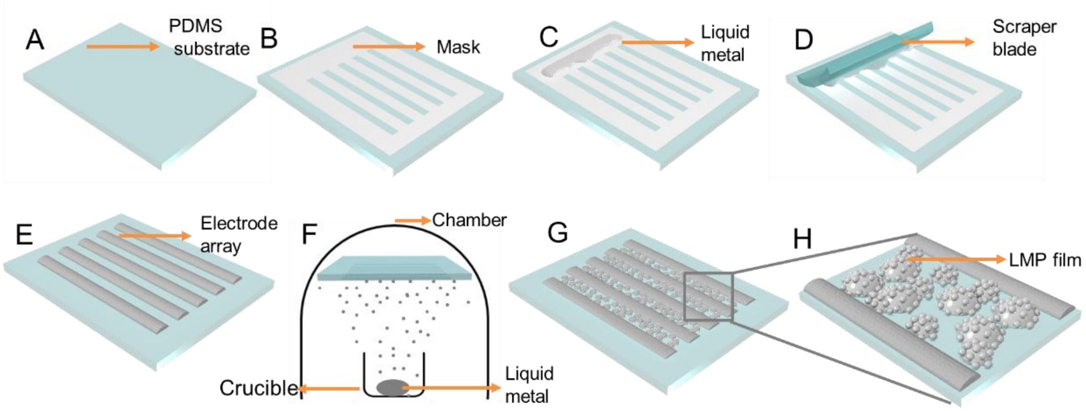

Figure S3. Schematic of the preparation process of damage sensor. **(A-E)**, The fabrication of electrode array on PDMS substrate. **(F-H)** Thermal deposition of liquid metal for LMP film.

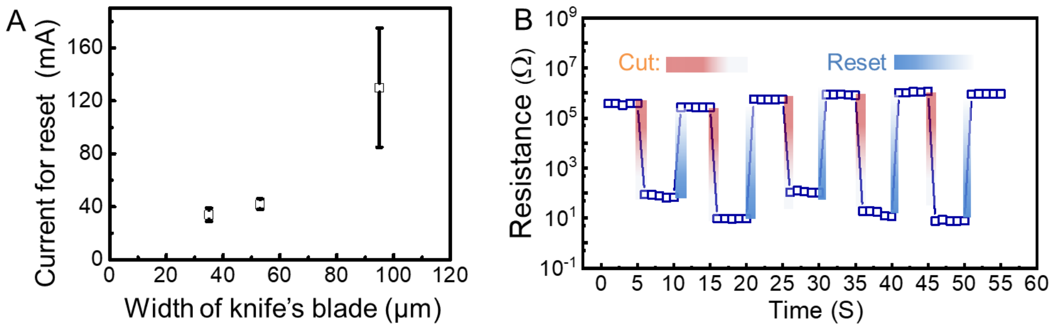

Figure S4. The reset properties of damage sensor cut by blade with different width. The resistance of damage sensor is at low stage after injury and can be reset to high resistance by applying current. The rest current increases when increase the width of blade. (b) The damage sensor is cut and reset for five cycles.

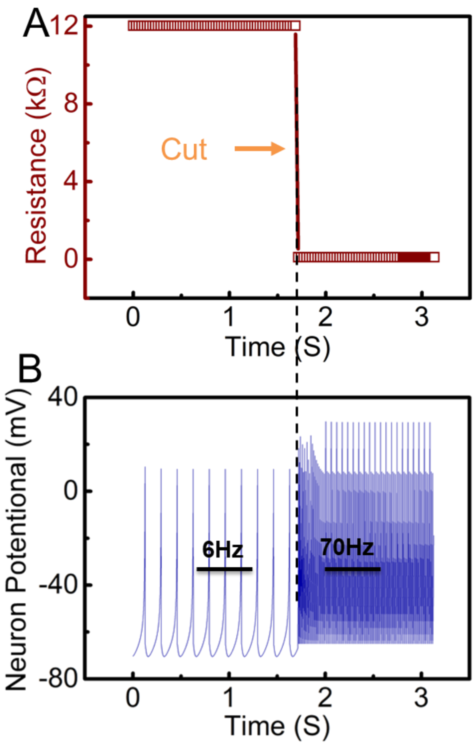

Figure S5. The properties of the MMPPS when being cut. **(A)**Before injured, R_{SMU} is 12 kΩ, which is decreased to less than 2.9 kΩ and induces pain. **(B)**, based on the Izhikevich neuron framework, the data in Figure A is transferred to the neuromorphic signal that the human nerve can recognize. Before injured, the neuromorphic signal following the mode of regular spiking (low frequency) and after injured it shifts to fast spiking (high frequency).

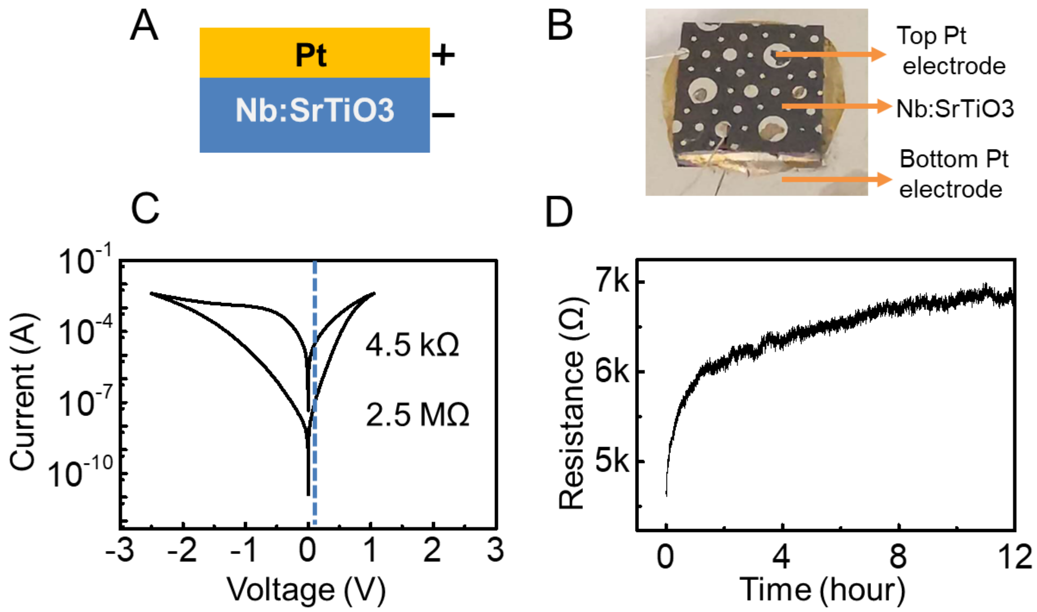

Figure S6. The properties of artificial synapse. **(A)** The diagram of the artificial synapse, which presents a simple Pt/Nb:$SrTiO_3$ heterojunction . **(B)** The optical image

of the artificial synapse. The black substrate is Nb: SrTiO3 single crystal and the Circles with metallic color are Pt electrodes. **(C)** When applying voltage from 0 V to 1.05 V, the resistance can be switch from high resistance state (MΩ, read by 0.1 V) to low resistance state (kΩ, read by 0.1 V). After this, when applying voltage from 0 V to -2.5 V, the resistance can be switched from low resistance state back to high resistance state. **(D)** The low resistance state of the artificial synapse will gradually shift from 4 kΩ to 6.8 kΩ in 12 hours.

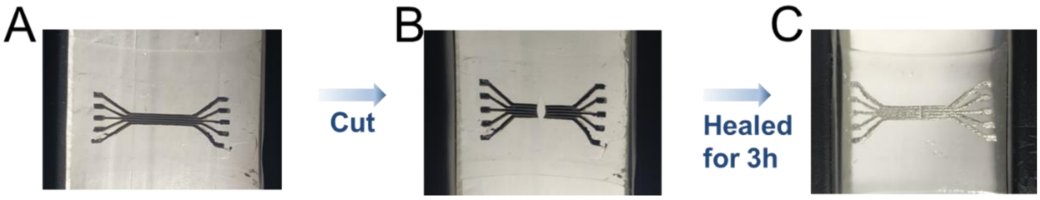

Figure S7. (A) damage sensor fabricated on self-healing PU and stretch the sample with the strain of 30%. (B) Cut the sample with a knife to generate damage. (C) After heated for 3 hours in 80 °C, the damage is healed.

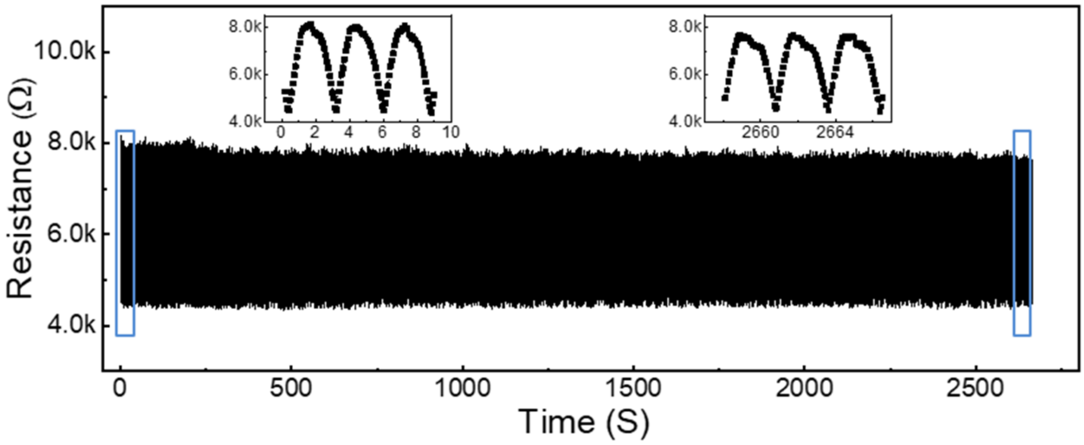

Figure S8. Stability of the pressure sensor tested for 1000 cycles, and the insets show the performance at the beginning and end of the test.

Neuromorphic models:

We used the Izhikevich neuron framework to process the current signal from the entire sensor system. Osborn *et al.* have effectively generated regular and fast spiking in real time by adjusting the model. Usually, the tactile signal corresponds to the regular-spiking of the neuron, and the pain corresponds to the fast-spiking of the neuron. Both types of neural signals can be achieved by adjusting the relevant parameters of the Izhikevich neuron framework (Prosthetics with neuromorphic

multilayered e-dermis). The two test units in Fig. 3A apply voltages and read corresponding currents. The current value is input into the MATLAB program as an input signal to calculate the corresponding neuron potential according to the Izhikevich neuron model. The function corresponding to this model is expressed in Equation 1, 2, and 3, where v is the membrane potential, and u is the refractory variable.

$$\frac{dv}{dt} = Av^2 + Bv + C - u + \frac{I}{RC_m} \quad (1)$$

$$\frac{du}{dt} = a(bv - u) \quad (2)$$

$$\text{if}(v \geq v_{th},)\text{then}\begin{cases} v = c \\ u = u + d \end{cases} \quad (3)$$

the values of the main constants are as follows: A = 0.04/Vs; B = 5/s; C = 140 V/s; Cm = 1 F; R = 1; b =0.2/s; c = – 65 mV; d = 8 mV/s; V_{th} = 30 mV. Through the value of a to determine the spiking mode.

The logic behind the implemented of this system is as follows:
First, we adopted 150 kPa as the threshold of normal skin to cause pain. Under this pressure, the resistance value of the pressure sensor is R0 (the example in Figure 3 is 2.9 kΩ). The resistance measured by SMU 1 is R1, and the current is I1. The resistance measured by SMU 2 is R2, and the current is I2. The switch between 0 and 1 of "flag" represents whether the skin is injured or not.
Set flag ==0; (system initialization)
if flag=0 (Working mode Before the injury)

if (R1>R0/2) (No injuries at this time)

If (R2>R0), then (regular spiking(I2), (There is no excessive pressure, the input signal of this model is I2).

else if (R2<R0), then (fast spiking(I2)), (At this time, the excessive pressure caused pain, and the input signal of this model was I2).

end

else if (R1<R0/2) (At this time is suffering from mechanical damage)

Then (fast spiking(I1)); (The decrease in damage-detection sensor 's resistance triggers pain; at this time, the input signal of the model is I1)

flag==1; (The skin has been injured)

activate artificial synapse; (Central sensitization mechanism is activated)

reset damage-detection sensor (damage-detection sensor is restored to a high-impedance state to guard against subsequent injuries)

end

else if(flag==1) (Working mode after injury)

if (R1< R0), (The pressure is too small to cause pain)

then (regular spiking(I1))

else if (R1< R0) (although the pressure is relatively slight, it causes pain)

then (fast spiking (I1))

end
